# P-595. Public Health Impact of RSVpreF Vaccination on Older Adult Disease Outcomes

**DOI:** 10.1093/ofid/ofae631.793

**Published:** 2025-01-29

**Authors:** Gonzalo Perez Marc, Daniel Eiras, Qin Jiang, Conrado J Llapur, Yasushi Fukushima, Mika Rämet, Louis J Bont, Nazreen Hussen, John Woodside, Tarek Mikati, Michael Patton, Agnieszka Zareba, Kumar Ilangovan, Kathy Schneider, Elena Kalinina, David Cooper, Kena A Swanson, Annaliesa S Anderson, Alejandra C Gurtman, Iona Munjal

**Affiliations:** i-trials, Buenos Aires, Ciudad Autonoma de Buenos Aires, Argentina; Pfizer, Inc., Pearl River, New York; Pfizer, Collegeville, Pennsylvania; Hospital del Niño Jesús, San Miguel de Tucuman, Tucuman, Argentina; Fukuwa clinic, Chuo-ku, Tokyo, Japan; Tampere University, Tampere, Pirkanmaa, Finland; University Medical Centre Utrecht, Utrecht, Utrecht, Netherlands; Netcare Lakeview Hospital, Benoni, Gauteng, South Africa; Pfizer, Collegeville, Pennsylvania; 3. Pfizer, Inc., Vaccine Research & Development, Pearl River, New York; Pfizer, Vaccine Research and Development, Hurley, England, United Kingdom; Pfizer, Collegeville, Pennsylvania; Vaccine Research and Development, Pfizer, USA, Raleigh, North Carolina; Pfizer, Collegeville, Pennsylvania; Pfizer, Collegeville, Pennsylvania; Pfizer, Collegeville, Pennsylvania; Pfizer, Collegeville, Pennsylvania; Pfizer, Collegeville, Pennsylvania; Pfizer, Collegeville, Pennsylvania; Pfizer Inc

## Abstract

**Background:**

The RSV Vaccine Efficacy Study in Older Adults Immunized Against RSV Disease (RENOIR) is a phase 3, multicenter, randomized, double-blinded, placebo-controlled study that demonstrated vaccine efficacy (VE) of bivalent RSVpreF vaccine to prevent lower respiratory tract illness (LRTI) caused by RSV in adults ≥ 60 years of age over two RSV seasons (NCT05035212). Here we present an analysis of the broader public health impact of bivalent RSVpreF vaccine.

Vaccine Efficacy and Public Health Impact of Vaccination with 1-Dose RSVpreF – mITT Efficacy Population

**Figure:**
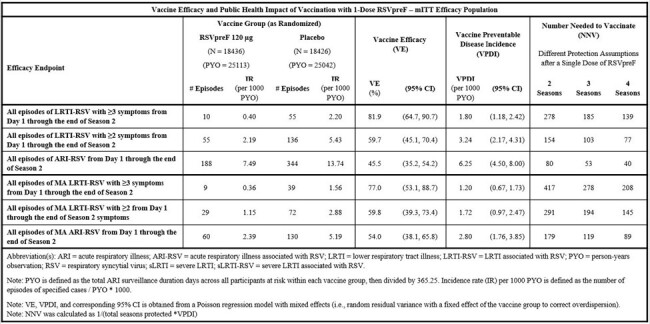

**Methods:**

To assess maximum public health impact of bivalent RSVpreF vaccination, all episodes of RSV-associated disease across a full surveillance period through two RSV seasons were included, utilizing a modified intention-to-treat (mITT) approach. This differs from prior efficacy estimations where first episodes of RSV-associated disease were used and assessed among the evaluable efficacy population. A Poisson regression model with mixed effects was used to estimate vaccine preventable disease incidence (VPDI), which was defined as control minus vaccinated group incidence. Numbers needed to vaccinate (NNV) were also estimated with different assumed durations of protection.

**Results:**

VE of bivalent RSVpreF vaccine was maintained across the full surveillance period (average 16.3 months) through two RSV seasons. VEs predictably increase as the RSV-associated disease severity increases (45.5%, 59.7%, and 81.9% for all episodes of ARI-RSV, LRTI-RSV with ≥ 2 symptoms, and LRTI-RSV with ≥ 3 symptoms, respectively). However, the VPDIs per 1000 person-years of observation (PYOs) decreased with increased severity of disease (6.25, 3.24, and 1.80, respectively). A similar trend was observed for medically attended (MA) events of RSV-associated disease (2.80, 1.72, and 1.20, respectively). Consequently, the NNV was lowest for episodes of ARI-RSV.

**Conclusion:**

This public health impact analysis of RSV-associated disease from a randomized controlled trial revealed a substantial reduction in RSV disease burden following older adult bivalent RSVpreF immunization based not only on demonstrated VE against more severe disease but also on VPDI against milder forms of RSV-associated disease, which represent a high proportion of overall RSV cases.

**Disclosures:**

**Gonzalo Perez Marc, MD**, Cassará: Expert Testimony|Cassará: Honoraria|Merck: Grant/Research Support|Moderna: Expert Testimony|Pfizer, Inc.: Advisor/Consultant|Pfizer, Inc.: Expert Testimony|Pfizer, Inc.: Grant/Research Support|Pfizer, Inc.: Clinical research|Sanofi: Grant/Research Support **Daniel Eiras, MD, MPH**, Pfizer, Inc.: Salary|Pfizer, Inc.: Stocks/Bonds (Public Company) **Qin Jiang, PhD**, Pfizer: Salary|Pfizer: Stocks/Bonds (Public Company) **Conrado J. Llapur, MD**, Pfizer, Inc: Grant/Research Support **John Woodside, PhD**, Pfizer: Stocks/Bonds (Public Company)|Pfizer, Inc.: salary|Pfizer, Inc.: Stocks/Bonds (Public Company) **Tarek Mikati, MD,MPH**, Pfizer, Inc.: salary|Pfizer, Inc.: Stocks/Bonds (Public Company) **Michael Patton, B.Sc.**, Pfizer: Employee|Pfizer: Stocks/Bonds (Public Company) **Agnieszka Zareba, MD PhD**, Pfizer, Inc.: salary|Pfizer, Inc.: Stocks/Bonds (Public Company) **Kumar Ilangovan, MD, MSPH, MMCi**, Pfizer, Inc.: salary|Pfizer, Inc.: Stocks/Bonds (Public Company) **Kathy Schneider, PhD**, Pfizer, Inc.: salary|Pfizer, Inc.: Stocks/Bonds (Public Company) **Elena Kalinina, PhD**, Pfizer, Inc.: Salary|Pfizer, Inc.: Stocks/Bonds (Public Company) **David Cooper, PhD**, Pfizer, Inc.: Employee|Pfizer, Inc.: Stocks/Bonds (Public Company) **Kena A. Swanson, Ph.D.**, Pfizer: Employee of Pfizer|Pfizer: Stocks/Bonds (Public Company) **Annaliesa S. Anderson, PhD**, Pfizer, Inc.: Employee|Pfizer, Inc.: Stocks/Bonds (Public Company) **Alejandra C. Gurtman, M.D.**, Pfizer, Inc.: Employee|Pfizer, Inc.: Stocks/Bonds (Public Company) **Iona Munjal, MD**, Pfizer: Salaried employee|Pfizer: Stocks/Bonds (Public Company)

